# What Is the Best Lens? Comparing the Resolution Power of Genome-Derived Markers and Standard Barcodes

**DOI:** 10.3390/microorganisms9020299

**Published:** 2021-02-02

**Authors:** Angela Conti, Laura Corte, Debora Casagrande Pierantoni, Vincent Robert, Gianluigi Cardinali

**Affiliations:** 1 Department of Pharmaceutical Sciences, University of Perugia, 06121 Perugia, Italy; angela.conti@studenti.unipg.it (A.C.); laura.corte@unipg.it (L.C.); deboracasagrandepierantoni@gmail.com (D.C.P.); 2 Westerdjik Institute for Biodiversity, 3584 Utrecht, The Netherlands; v.robert@wi.knaw.nl; 3 CEMIN Excellence Research Centre, 06123 Perugia, Italy

**Keywords:** species delimitation, taxonomy, yeast, marker, ITS, LSU, *RPB1-2*, *ACT1*, *TEF1α*, barcoding

## Abstract

Fungal species delimitation was traditionally carried out with multicopy ribosomal RNA (rRNA) genes, principally for their ease of amplification. Since the efficacy of these markers has been questioned, single-copy protein-encoding genes have been proposed alone or in combination for Multi-Locus Sequence Typing (MLST). In this context, the role of the many sequences obtained with Next-Generation Sequencing (NGS) techniques, in both genomics and metagenomics, further pushes toward an analysis of the efficacy of NGS-derived markers and of the metrics to evaluate the marker efficacy in discriminating fungal species. This paper aims at proposing *MeTRe* (Mean Taxonomic Resolution), a novel index that could be used both for measuring marker efficacy and for assessing the actual resolution (i.e., the level of separation) between species obtained with different markers or their combinations. In this paper, we described and then employed this index to compare the efficacy of two rRNAs and four single-copy markers obtained from public databases as both an amplicon-based approach and genome-derived sequences. Two different groups of species were used, one with a pathogenic species of *Candida* that was characterized by relatively well-separated taxa, whereas the other, comprising some relevant species of the *sensu stricto* group of the genus *Saccharomyces*, included close species and interspecific hybrids. The results showed the ability of *MeTRe* to evaluate marker efficacy in general and genome-derived markers specifically.

## 1. Introduction

The advent of Next-Generation Sequencing (NGS) fostered genomic studies transforming genomics from highly specialized, expensive, community-based work [1] into a routine activity with several opportunities that go well beyond the study of genomes, per se [2]. Among the various possibilities offered, there is a better understanding of genome evolution and its usage in phylogeny and taxonomy [3,4,5]. In fact, genome analysis could replace the practice of single-locus markers, or the more accurate multi-locus analysis, for the definition of species boundaries and for the identification of strains at the species level [6,7,8,9,10,11,12].

In order to achieve this goal, the tools and approaches must be set up properly, because more data do not necessarily mean an increase in resolving power [13]. The long-lasting problem is an appropriate and applicable species concept [14] among the approaches that are currently used in both bacterial and fungal biology, including phylogeny, genetic segregation and phenetics [15]. Among these three approaches, phylogeny can be used for classification and for identification, although it is much more computer-intensive then phenetics. At the beginning of the sequencing era, DNA/DNA hybridization was still considered the “optimal method for measuring the degree of relatedness between highly related organisms” [11], and the same fixed homology thresholds were proposed to separate all species within the kingdom or the superkingdom. The interest was to establish whether two strains belonged to the same species using the same approach as the DNA/DNA-related analyses, resulting in sentences like: “at sequence homology values below about 97.5%, it is unlikely that two organisms have more than 60 to 70% DNA similarity and hence that they are related at the species level.” [11]. The same concept was taken over in a seminal paper on ascomycetous yeasts: “Conspecific strains generally had fewer than 1% nucleotide substitutions in this domain, whereas biological species were separated by greater than this number of substitutions, thus providing an empirical means for recognizing species.” [8]. These thresholds are obviously somewhat arbitrary; moreover, it is not even necessarily true that all taxa can be separated by applying the same distance reference values, although some consensus can be found when massive analyses are carried out [16].

The use of distances with fixed thresholds poses the problem of the relative distance within the species and the distance among closer species [17]. In general, problems can be expected with larger species, i.e., those with more marker variability, mainly when the intraspecific distance is equal to or larger than the threshold or the actual interspecific distances. Another aspect is the quality of the DNA sequence used; in fact, not all databases are appropriately curated, and only a portion of the marker sequences are carefully checked before publishing them in the appropriate repository [18,19]. These problems arise equally with traditional sequences obtained from single-strain DNA with an amplicon-based approach and with genomic-derived sequences achieved with some NGS platform, most often from short reads assembled or mapped to a reference database. Although apparently similar, the sequences obtained with these two approaches may present significant differences, especially when the loci of interest are highly repeated, as in the case of the DNA encoding the ribosomal RNA (rRNA) in eukaryotes like fungi [20,21,22,23]. The multicopy nature of rDNA implies some level of heterogeneity amongst the various copies, in spite of homogenizing mechanisms of concerted evolution by gene conversion or by putative birth-and-death mechanisms [24,25]. Whatever the reason, these markers give different outputs with Sanger or NGS; in fact, the former reports a sort of “mean sequence” in which the polymorphic sites are reduced to the most abundant nucleotide; conversely, the latter reports every single variation in the various reads spanning over the polymorphic sites [26].

The differences between the two approaches have been also investigated by cloning a portion of the DNA region and by sequencing the various clones separately [27]. Both NGS and cloning confirmed the presence of polymorphisms that obviously play a role when multicopy markers are taken from genomes. Meanwhile the evidence of the high internal variability of rDNA was accumulated; there was an active research of novel single-copy protein-encoding loci to be used as markers singularly or in combinations in the frame of multigene (or multi-locus) phylogenetic analyses [9,28]. Although these markers are quite promising in terms of fungal phylogenetic signal and taxonomic resolution power, the problem of their PCR amplification is quite difficult to overcome to the point that some of these markers require a precise and complex multistep amplification strategy with several regions of the same marker sequenced separately. Moreover, not many taxa have been analyzed for the presence and performance of such genes, so now their utility is questionable, although their potential remains high. In a genomic scenario, these single-copy markers would greatly benefit from NGS technology, overcoming the problems of difficult amplification and cumbersome sequencing experienced with Sanger sequencing [9,28].

Altogether, this rapid overview on the currently proposed markers shows that the passage from Sanger sequencing of individual strains to NGS of the genome is likely to produce some problems related to rRNA multicopy genes and to alleviate those of the single-copy markers.

In order to tackle this problem, the novel metric Mean Taxonomic Resolution (*MeTRe*) is proposed as a tool to determine the efficacy of markers. Using this index and other tools, the main goals of this paper are (i) to compare the performances of the standard barcodes (Internal Transcribed Spacer (*ITS*) and Large ribosomal subunit (*LSU*)) obtained with an amplicon-based approach and then sequenced with either Sanger or NGS techniques and those retrieved directly from complete genomes and (ii) to assess the ability of single-copy genes to be used as barcodes.

## 2. Materials and Methods

### 2.1. Collection of Sequences

All the *Saccharomyces sensu stricto* and *Candida* genomes were obtained from the National Center for Biotechnology Information (NCBI). A total of 58 genomic FASTA files for *Saccharomyces* and 26 genomic FASTA files for *Candida* were retrieved from the Assembly database using the filter [Organism] to get exclusive data from a determined species. Due to the limited number of available genomes in the NCBI, the species involved in this study were: *S. bayanus*, *S. cerevisiae*, *S. kudriavzevii*, *S. paradoxus*, *S. pastorianus* and *S. uvarum* for the *Saccharomyces sensu stricto* complex, while the analysis was restricted to *C. albicans*, *C. auris*, *C. glabrata*, *C. metapsilosis*, *C. otrhopsilosis*, *C. parapsilosis* and *C. tropicalis* for the *Candida* genus. To have a comparable number of genomes per species, 10 genomes per *Saccharomyces* species were randomly chosen among all the results, and 4 genomes for each *Candida* species were collected. Accession numbers of these genomes are listed in Table 1. Sequences of six commonly used markers were taken from the YeastIP database (http://genome.jouy.inra.fr/yeastip/) and the NCBI. These markers were used as probes for discovering the exact position of such sequences within the genome. Probe sequences used in this study were: Actin (*ACT1*), Internal Transcribed Spacer (*ITS*), Large ribosomal subunit (*LSU*), Translational Elongation Factor 1-alpha (*TEF1α*), RNA polymerase II Largest subunit (*RPB1*) and RNA polymerase II second-largest subunit (*RPB2*). For each species of the *Saccharomyces sensu stricto* complex, only type strain sequences were considered, while for the *Candida* genus, non-type strain sequences were also used where the type strain sequences were not present in any database. The probe sequences used in this study are reported in Table 2.

### 2.2. Capturing Markers from Genomes

Probe sequences (Table 2) were aligned to the genomes using the *nucmer* packages included in the MUMmer system [29]. It is an anchor-based multiple alignment that allows two multi-FASTA inputs to be aligned by using Maximal Unique Matches (MUMs), which are matches that occur once in each genome. To use all anchor matches, regardless of their uniqueness, the option *-maxmatch* was used as follows:

Nucmer *-maxmatch* [input_genome.fna] [probe_sequences.fasta] *-p* [output]

The output produced is a .delta file that was converted into a text file using the function *show-coords*, a submodule of the MUMmer package. The options -c (to show the coverage percentage of the alignment) and -l (to include sequence length information in the output) are also included. Another option used in the command is -r, which allows to sort output lines by reference IDs.

The coordinates obtained in the text file were used to select a specific region of the genomes with the utility *samtools faidx* of the package SAMtools. With this function, a subsequence is extracted from the indexed reference sequences. Each marker considered in the study was extracted from all the genomes, using the coordinates with tags [S1] and [E1] in the text file, which referred to the start and the end of the alignment region in the reference sequence. A total of 348 sequences for the *Saccharomyces sensu stricto* complex, and 145 sequences for the *Candida* genus were retrieved and stored in a separate FASTA file.

### 2.3. Alignment and Data Analysis

All the sequences of a specific marker, retrieved from the genomes of the species in the analysis, were merged in a FASTA file, for a total of 12 files (6 for *Saccharomyces* and 6 for *Candida*). The files also contained the sequences of the type strain for each specific marker. Similarly, amplicon-based sequences of ITS and LSU, for both the *Saccharomyces sensu stricto* complex and *Candida* genus, were collected in 2 different files (Supplementary Table S1). These sequences were retrieved from the NCBI database.

Each file was used for carrying out multiple alignment with the algorithm ClustalW in MEGA 7 [30]. The parameters chosen for the alignment were: Gap Opening Penalty 15 and Gap Extension Penalty 6.66 for both pairwise and multiple alignment, while the transition weight was considered equal to 0.3. The same parameters were applied for the multiple alignment of amplicon-based sequences. For each alignment, the distance matrix and Neighbor-Joining tree were calculated with the functions *dist.dna* and *nj*, respectively, of the ape package (v. 5.4.1) in R ( version 4.0.3, Platform: x86). The option used for distance matrixes was a “raw” model, which is the proportion of sites that differ between each pair of sequences. A Mantel test was carried out to compare the results obtained from type strain genomic markers against the corresponding amplicon-based sequences, with the function *mantel.test* (package Vegan, v. 2.5.6) that computes Mantel’s permutation test for the similarity of two matrices. Additionally, the trees obtained from the *ITS*, *LSU* and *TEF-1α* genomic sequences and amplicon-based sequences, separately, were compared with the package *phylogram* (v 2.1.0) in R.

### 2.4. Inter and Intra-Group Distances Analysis

Marker sequences retrieved from genomes were used to calculate both the distances (p-distances algorithm, MEGA 7) among the strains of the same species and among the different species. The functions *Between Groups Mean Distances* and *Within Groups Mean Distances* in MEGA 7 were used. The first algorithm computes the average distances between groups of taxa, which are the arithmetic means of all pairwise distances between two groups in the inter-group comparisons. While the *Within Groups Mean Distances* are arithmetic means of all individual pairwise distances between taxa within a group. For both analyses, the parameters used were p-distances as the substitutions model, which included Transition + Transversion and uniform rates for sequence evolution.

### 2.5. Calculation of MeTRe

Distances, obtained with MEGA 7 functions, represented the input of a Macro, written in MS Excel, to compute the resolution of each marker. The resolution index, called *MeTRe*, was calculated as the ratio between the value of interspecies distance and the sum of intraspecies distances of the two species compared as indicated in the two formulas:

(1) $$MeTRe_{i-j}=\frac{{Dist}_{i-j}}{\left({Dist}_{i}+\;{Dist}_{j}\right)}$$

(2) $$MeTRe_{i-j}=\;\frac{{Dist}_{i-j}}{Th}$$

In which the pedix (*i − j*) indicates the *MeTRe* deriving from the comparison of the *i*th with the *j*th species, and similarly, the *Dist_i−j_* indicates the intraspecific distance, whereas *Di* and *Dj* indicate the mean internal variability of the *i*th and *j*th species. In Formula (2), the mean internal variability is substituted with the sum of the two-half threshold distance (*Th*) accepted for the marker in use.

In the square matrix reporting *MeTRe* data, the descending diagonal reported the intraspecific distances divided by themselves and, therefore, = 1. The intraspecific distances are the mean internal variability of the species (Formula (1)); alternatively, it can be half of the distance threshold accepted for that marker (Formula (2)). The upper and lower triangular matrixes contained the values of the Mean Taxonomic Resolution (*MeTRe*), which indicate no resolution with *MeTRe* ≤ 1 and resolution with *MeTRe* > 1. The possibility of using two different types of data for intraspecific distances was tested with *ITS* and *LSU* by calculating two different *MeTRe* matrices, one with values obtained with the algorithm *Within Groups Mean Distances* in MEGA 7, while the other with the threshold values, i.e., 0.7 for *ITS* and 0.5 for *LSU* (half of the accepted taxonomic thresholds) [16].

## 3. Results

### 3.1. Experimental Design

The present work aims at comparing the distances among strains with the variability within the species to evaluate the different taxonomic resolutions obtainable with various markers. The species and the strains selected respond to the double criterion of taxonomic models accommodating relatively close species and of species with enough genomes published from which taxonomic markers could be obtained. Using these two criteria, we selected several species belonging to the *Saccharomyces* genus and to the group of the pathogenic species of the *Candida genus*.

Along with *LSU*, *ITS* is well-known and used as “universal barcoding marker” for fungi [31], with several amplicon-based sequences available in public databases, some of which are under active curation [19]. Since there is not, to our knowledge, a repository of *LSU* or *ITS* sequences derived from genomes, we decided to retrieve them directly from published genomes. All distances analyses were carried out considering the amplicon-based sequence of the type strain as the reference sequence from which both intra- and interspecific distances were obtained and evaluated.

### 3.2. Distance Analysis among Saccharomyces Species

**Figure 1:**
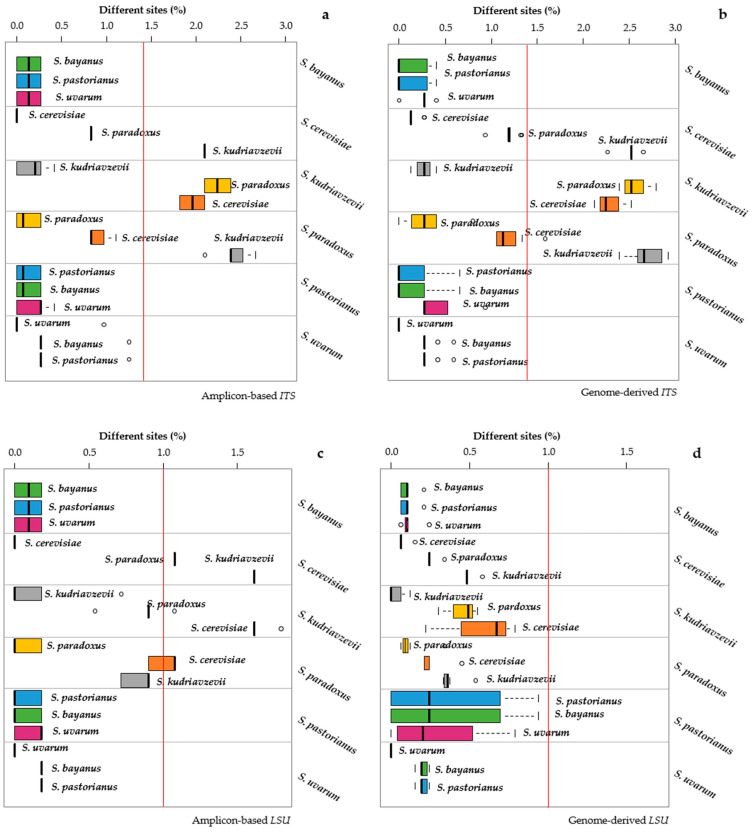
Distances among and within the *Saccharomyces* species. Distances obtained with standard and genomic Internal Transcribed Spacer (*ITS*) (expressed as % of substitution in comparison to the type strain) are displayed in panels (**a**,**b**), respectively. The standard and genomic distances from Large ribosomal subunit (*LSU*) markers are reported in panels (**c**,**d**), respectively. Each subpanel reports the distances of the strains belonging to the species under study (indicated on the right of the subpanel) with the sequences of the type strains of the three closest species (indicated close to the box). For instance, the topmost subpanel of (**a**) reports the intra- and interspecific distances of *S. bayanus* from the sequences of the *S. bayanus*, *S. pastorianus* and *S. uvarum* type strains. The width of each box represents the variability of the distances of the strains of each species from the type strains of the three closest species. The internal variability of each species is indicated by the topmost box of each subpanel. The distances among the means of the species are displayed as the distances between the vertical l, thick segments within the boxes.

The distance analysis with *ITS* and *LSU* sequences, considering both standard barcodes and genome-derived sequences, was carried out by comparing all the strain sequences with the sequence of the type strain of each species under investigation. The results were aggregated by species, reporting the distances within and among groups of the three closest species, eliminating the others for the clarity of presentation (Figure 1).

The analysis in groups of three species also responded to the problem of considering the ability of each marker to separate from the closest, with the farthest not representing a taxonomic problem. The accepted thresholds [8,16] of 1.4% for *ITS* and 1% for *LSU* were adopted and reported as a horizontal red line. Therefore, there is poor separation for the species falling in the area below the red line. A preliminary analysis showed that most cases with a poor separation were circumscribed to groups of three species. Using *ITS* as a marker, both the standard and genomic sequences of *S. bayanus* were not separated from *S. pastorianus* and *S. uvarum* type strains. Although the variability within these three species was not particularly high, the mean distances among them were little, and no separation could be obtained (Figure 1a,b). *S. cerevisiae* and *S. paradoxus* showed a relatively low internal variability, but the intraspecific distance was lower than the adopted threshold, and they were, therefore, poorly separated. *S. paradoxus* sequences showed a worse situation compared to *S. cerevisiae*; in fact, the interspecific variability with *S. cerevisiae* and *S. paradoxus* type strains was higher than that of *S. cerevisiae* with both genomic and amplicon-based sequences, although the latter had a slightly better performance (Figure 1a,b). The sequences of *S. uvarum* showed low internal variability with both genomic and standard markers, whereas the internal variability was large in *S. pastorianus*, which showed no separation with the two closest species. 

With the *LSU* marker (Figure 1c,d), most of the results were like those obtained with ITS, with some remarkable differences. Firstly, the genomic *LSU* marker failed to separate the species over the 1% threshold; in fact, the largest distances recorded using those markers were around 0.9%. In general, the internal genomic distances were larger than those obtained with amplicon-based sequencing. Then, the internal variability of the sequences retrieved from *S. pastorianus* genomes was extremely large when the comparison was carried out with the *S. pastorianus*, *S. bayanus* and *S. uvarum* type strain sequences. On the contrary, when using the sequences obtained from *S. bayanus* and *S. uvarum* genomes, the internal variability was comparable with the variability of the *LSU* standard sequences.

### 3.3. Distance Analysis among the Pathogenic Candida Species

**Figure 2:**
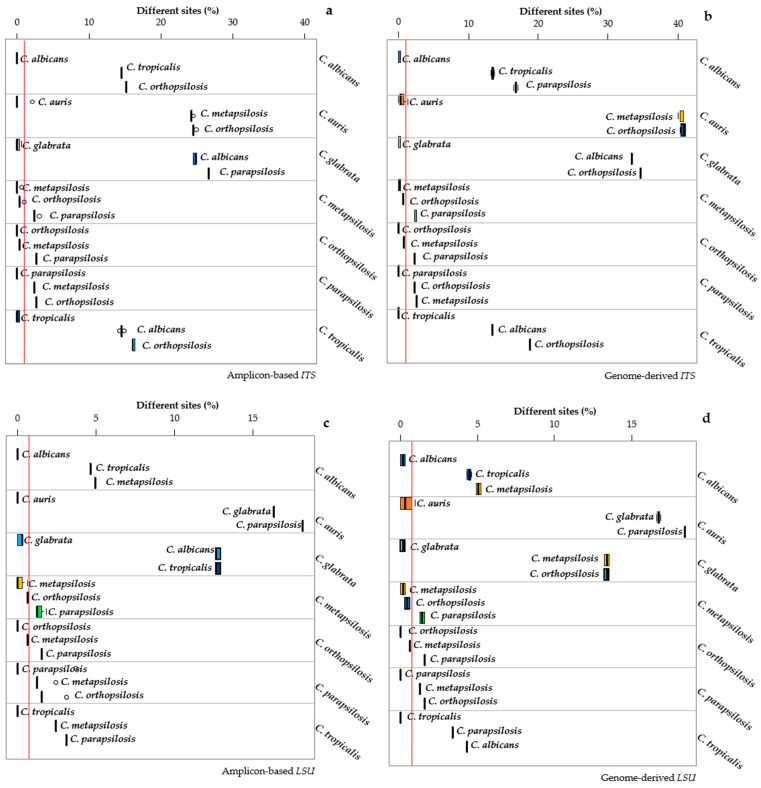
Distances among and within *Candida* species. The distances obtained with standard and genomic *ITS* are displayed in panels (**a**,**b**), respectively. The standard and genomic distances from the *LSU* markers are reported in panels (**c**,**d**). Each subpanel reports the distances of the strains belonging to the species under study (indicated on the right of the subpanel) with the sequences of the type strains of the three closest species (indicated close to the box). For instance, the topmost subpanel of (**a**), reports the intra and interspecific distances of *C. albicans* from the sequences of the *C. albicans*, *C. tropicalis* and *C. orthopsilosis* type strains. The width of each box represents the variability of the distances of the strains of each species from the type strains of the three closest species. The internal variability of each species is indicated by the topmost box of each subpanel. The distances among the means of the species are displayed as the distances between the vertical thick segments within the boxes.

The same analyses described above were carried out with the pathogenic species of the genus *Candida*—among which, three species (*C. parapsilosis*, *C. metapsilosis* and *C. orthopsilosis*) were derived from the splitting of the former *C. parapsilosis* species [32]. Furthermore, *C. glabrata* is phylogenetically different from the others, stemming from whole-genome duplication [33]. The internal distances of all *Candida* species (as resulting from the datasets employed) were much smaller than the distances among the various species. In fact, interspecific distances were up to 25% and 40% for the standard and genomic ITS and up to 15% for both types of *LSU* sequences. *C. metapsilosis* could not be separated from *C. orthopsilosis* with any of the two markers, irrespective of the sequencing method used (Figure 2). In general, the internal variability of the genomic sequences was higher than that of the corresponding amplicon-based.

### 3.4. Proposal of Mean Taxonomic Resolution (MeTRe) as a Novel Metric to Determine Marker Efficiency

The distance analysis presented in the above paragraphs takes into consideration inter- and intraspecific distances qualitatively, making their comparison rather complex. In order to overcome this problem, the Mean Taxonomic Resolution (*MeTRe*) metrics were calculated as the ratio between the mean distance among a pair of species and the sum of internal variability of the two species. It is therefore “1” when a species is compared with itself, less than 1 when the two species are not well-separated and higher than 1 when the two species are well-discriminated by the marker in use. In other words, *MeTRe* defines the distance between two species using their internal variability as the unit. *MeTRe* can be considered a tool to define the real separation between species or as a metric to compare the efficacy or markers in separating species. The present paper uses the latter approach to define and compare quantitatively the effectiveness of amplicon-based and genome-derived markers. Since *ITS* and *LSU* are well-known markers, the internal mean variability of the species was set at 0.7% and 0.5%, respectively, i.e., at half the level of the threshold suggested for these markers [8,16]. 

**Figure 3:**
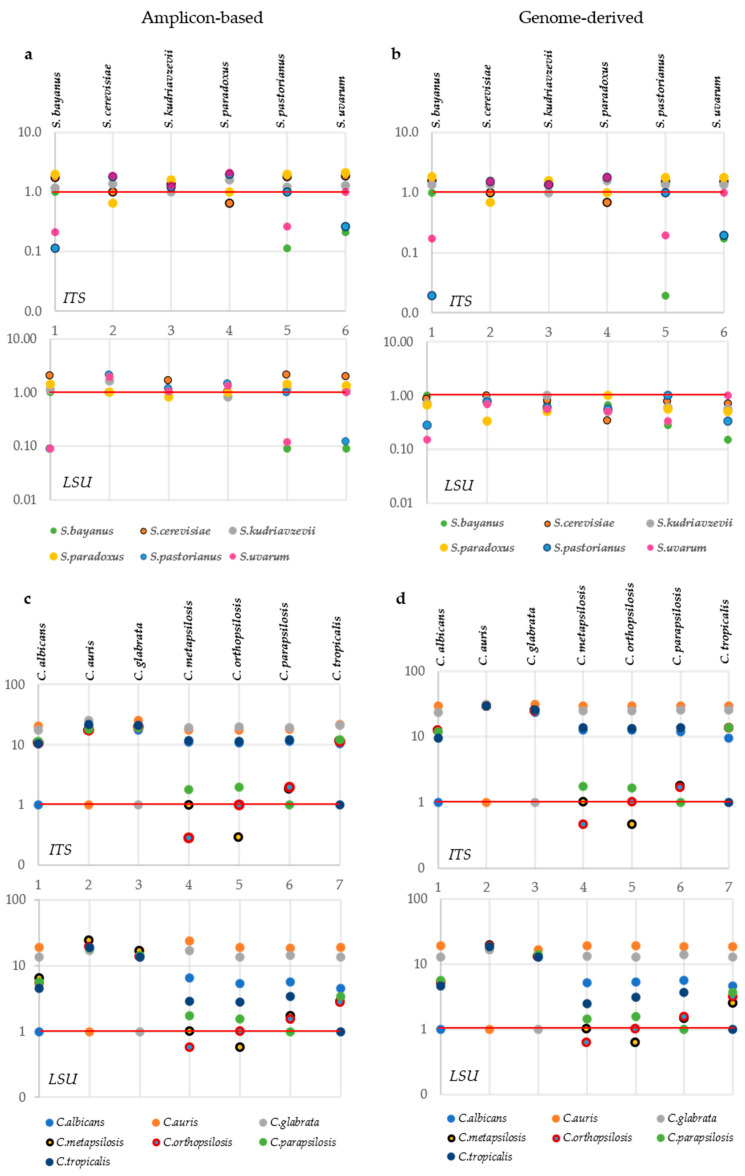
Mean Taxonomic Resolution (*MeTRe*) among and within the *Saccharomyces* and *Candida* species. Resolution calculated for *Saccharomyces* standard and genomic *ITS* and *LSU* are displayed in panels (**a**,**b**), respectively. Amplicon-based and genomic resolution of the *Candida ITS* and *LSU* markers are reported in panels (**c**,**d**). Each column shows the resolution of the species positioned in a red line (reported above each column) from the other species under analysis. The species placed below the red line were not resolved by the marker from the species reported above each column. Values of *MeTRe* in log scale are reported on the y-axis, while numbers on the x-axis correspond to the species involved in the study (and are depicted at the beginning of each subpanel).

The *MeTRe* analysis for the *ITS* and *LSU* markers derived the standard or genomic sequences, following the same experimental scheme presented in the previous paragraphs, but it allowed to accommodate in the same graph the resolution of each species in comparison with all other species considered. A comparison of the corresponding panels in Figure 1 and Figure 2 vs. Figure 3 showed that all cases without a separation according to the distance analysis were confirmed by low *MeTRe* values, indicating that the metric gives a faithful representation of the overall taxonomic relationships.

More importantly, *MeTRe* allows to define the overall and specific delimitation efficiency of the markers, thus allowing a direct comparison. For instance, in *Saccharomyces*, ITS showed an average *MeTRe* of 1.24 and 1.17 for the amplicon-based and the genomic sequences, respectively, whereas, for *LSU*, these values were 1.14 and 0.63. These figures indicate that the *ITS* performed slightly better when considering the amplicon-based sequences rather than the genome-derived markers. On the contrary, genome-derived *LSU* had a much worse performance than the corresponding amplicon-based. An average *MeTRe* of around 1 for the *ITS* is, in any case, an indication of a rather poor performance of the marker for the species under analysis. In fact, the distance among species is only slightly more than the distance within the species, indicating cases in which there is little, if any, resolution. When the same analysis was carried out among the *Candida* species considered, *ITS* had 12.4 and 16.2 average *MeTRe* and *LSU* 8.6 and 9.2 for the standard and genome sequences, respectively. These data allowed to define quantitatively that the performance of *ITS* is better than that of *LSU* in both taxonomic models. On the other hand, *MeTRe* allowed to rapidly define that, in the *Candida* model adopted for this study, the separation among species was roughly ten times their internal variability. These figures indicated that high *MeTRe* may not mean much if the species considered are very distant—in which case, there is not a real taxonomic problem. Conversely, *MeTRe* was interesting when comparing two close known species, i.e., where species delimitation becomes a real taxonomic problem. 

*MeTRe* can be calculated using the standard internal variability values, as described above, or the actual variability displayed by the strains of each species. The latter system offers a more realistic evaluation of the actual resolution of the markers among the species and produces better results when large numbers of sequences are used. When using the latter approach with the same sequences, the mean *MeTRe* of the *Saccharomyces* species were 5.04, 9.33, 14.97 and 7.85, respectively, for the standard and genomic *ITS* and standard and genomic *LSU*. Similarly, for *Candida*, the four mean *MeTRe* were 39.34, 52.70, 33.17 and 17.49, again larger than those produced with fixed thresholds. These figures are much larger than those found using a fixed internal variability, as above, because the actual variability of the strains within each species was lower than the 0.7% and 0.5% chosen as half of the *ITS* and *LSU* threshold. When the internal variability of a species was relatively high, as for *S. pastorianus* genomic *LSU*, the *MeTRe* values decreased, and the average *MeTRe* of this species was 0.88, whereas those of the other species ranged from 6.6 and 12.3.

Both systems can be used to evaluate the marker efficacy if the consensus threshold values are known, whereas the usage of the actual internal variability is the only approach when new markers are studied, as in the cases presented in the subsequent part of this paper.

### 3.5. Single-Copy Markers from Genomes

Single-copy protein-encoding genes are known to be superior to rRNA genes in phylogeny and species delimitations but are also afflicted by problems in amplification and by the consequent lack of wide libraries [31]. Nevertheless, some studies were carried out using these genes with many fungal species and genera [9,28]. The possibility to retrieve these sequences from genomes is an appealing perspective to accomplish a multigene phylogenetic analysis and accurate delimitation of the fungal species. Furthermore, the increasing rapidity and decreasing costs of the genome sequencing procedure might lead to using these markers for identification as well. In order to understand their efficacy, we conducted the same analyses described above with a series of single-copy protein encoding genes obtained from the same genomes. 

*S. pastorianus* genomes harbor two types of the single-copy genes: one similar to *S. bayanus* and one to *S. cerevisiae*; the former was marked with a B and the latter with a C.

**Figure 4:**
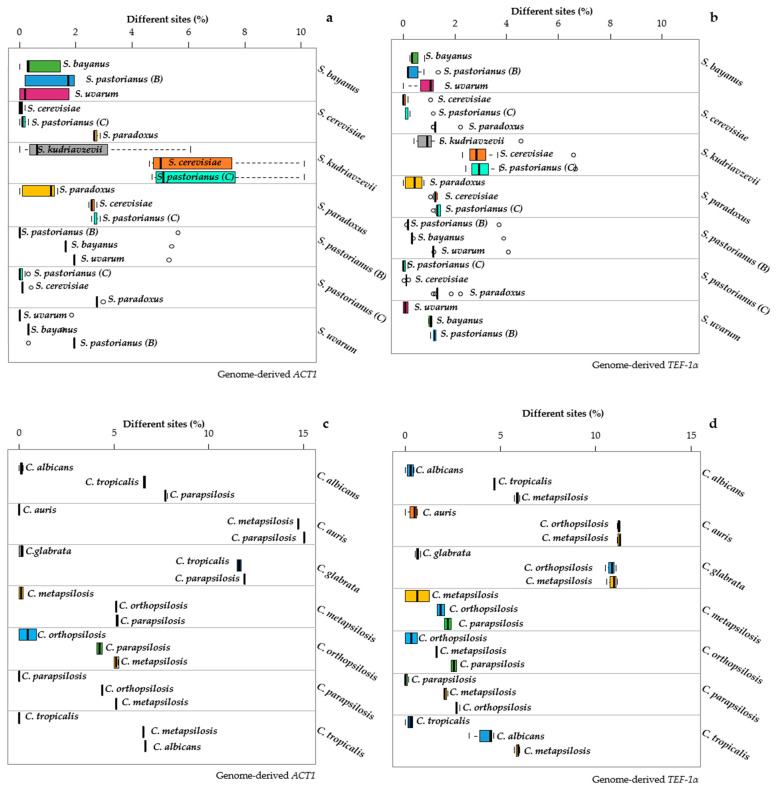
Distance among and within the *Candida* and *Saccharomyces* species. Distance calculated for the genomic Actin (*ACT1*) and genomic Translational Elongation Factor 1-alpha (*TEF1-α*) of *Saccharomyces* are displayed in panels (**a**,**b**), respectively. Distance calculated for the genomic *ACT1* and genomic *TEF1-α* of *Candida* are reported in panels (**c**,**d**). Each subpanel reports the distances of the strains belonging to the species under study (indicated on the right of the subpanel) with the sequences of the type strain of the three closest species (indicated close to the box). The width of each box represents the variability of the distances of the strains of each species from the type strains of the three closest species. The internal variability of each species is indicated by the topmost box of each subpanel. The distances among the means of the species are displayed as the distances between the vertical thick segments within the boxes.

*ACT1* sequences showed a large internal variability in *S. bayanus*, *S. paradoxus* and *S. kudriavzevii* (Figure 4a).

*S. kudriavzevii* showed a relatively low average (0.25%) but a large range (>3%) of the distances between its strains and the type, confirmed by a large variability when these strains are compared with the types of *S. pastorianus* and *S. cerevisiae*. This could be due either to the presence of incorrectly identified strains or to the poor quality of the genomes. A similar case was present with the *ACT1* of *C. orthopsilosis* (Figure 4c), where the strains showed variability only against their own type strain, whereas they showed larger distances with the types of *C. parapsilosis* and *C. metapsilosis.* These two cases showed that, when the distances between species are large, even a high internal variability does not limit the species separation.

In general, the variability shown by this marker is much lower than the interspecific distances, and a good separation of the species can be obtained, except for *S. bayanus, S. pastorianus (B)* and *S. uvarum* and for *S. cerevisiae* and *S. pastorianus (C)* (Figure 4a). There was registered a strong separation between the copy “B” and “C” retrieved from the genome of *S. pastorianus*, with the former belonging to the group of *S. bayanus* and *S. uvarum* and the latter more similar to the group of *S. cerevisiae* and *S. paradoxus* (Figure 4a).

All species of *Candida* were well-separated, thanks to larger distances among the species up to 15% vs. the 10% in *Saccharomyces.*

*TEF1a* showed similar patterns to *ACT1*, with all *Candida* species well-separated and with relatively low internal variability (Figure 4d) and the lack of separation in *Saccharomyces* reported above for *ACT1*.

**Figure 5:**
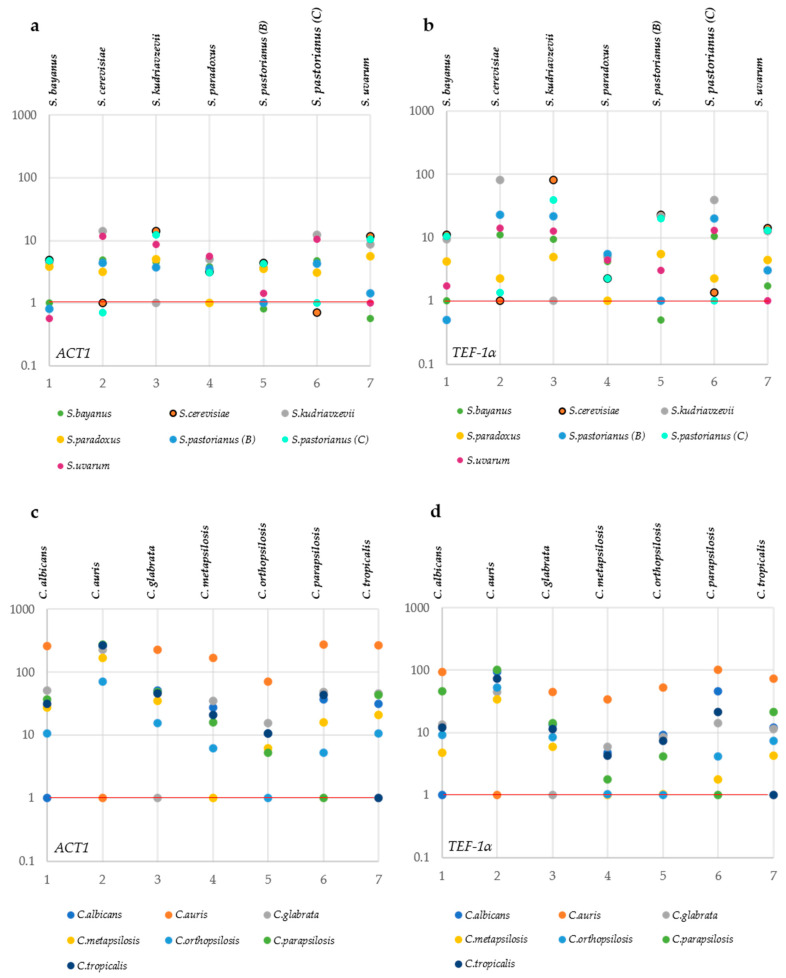
*MeTRe* among and within the *Saccharomyces* and *Candida* species for *ACT1* and *TEF1-α.* Resolutions calculated for the genomic *ACT1* and genomic *TEF1-α* of *Saccharomyces* are displayed in panels (**a**,**b**), respectively. Genomic *ACT1* and genomic *TEF1-α* of *Candida* are reported in panels (**c**,**d**). Each column shows the resolution of the species positioned in a red line (reported above each column) from the other species under analysis. The species placed below the red line have no resolution with the species reported above each column. The values of *MeTRe* in log scale are reported on the y-axis, while the numbers on the x-axis correspond to the species involved in the study (and depicted at the beginning of each subpanel).

The *MeTRe* analysis displayed these situations in a straightforward way. The only species grouped below the level of *MeTRe* = 1 were the groups B and C with both *ACT1* and *TEF1a* (Figure 5). Furthermore, *MeTRe* could show that, within B group, the *S. bayanus* strains were not separated from *S. pastorianus (B)* and *S. uvarum*, whereas the *S. uvarum* strains were separated from the *S. pastorianus (B)* sequence and not from *S. bayanus* (Figure 5a).

**Figure 6:**
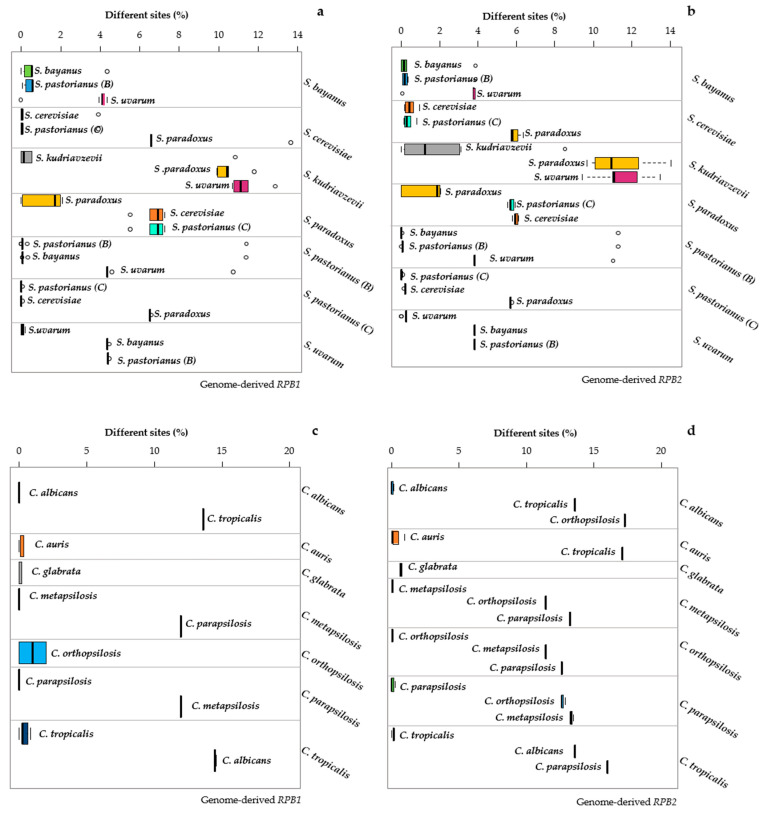
Distance among and within the *Candida* and *Saccharomyces* species. Resolutions calculated for genomic *RPB1* and genomic *RPB2* of *Saccharomyces* are displayed in panels (**a**,**b**), respectively. Genomic *RPB1* and genomic *RPB2* of *Candida* are reported in panels (**c**,**d**). Each subpanel reports the distances of the strains belonging to the species under study (indicated on the right of the subpanel) with the sequences of the type strain of the three closest species (indicated close to the box). The width of each box represents the variability of the distances of the strains of each species from the type strains of the three closest species. The internal variability of each species is indicated by the topmost box of each subpanel. The distances among the means of the species are displayed as the distances between the vertical thick segments within the boxes.

Similarly, the sequences of *S. pastorianus* were separated from those of *S. uvarum* but not from *S. bayanus*. These cases showed that *MeTRe* can dissect situations difficult to disentangle even with an accurate distance analysis. Moreover, the possibility of using *MeTRe* = 1 as a discriminant allowed to define the cases of species separation without the use of an arbitrary distance threshold, because it indicates the cases in which strains at the borders of the two species would be equally distant from the two type strains. *MeTRe* values obtained from the distances calculated with *RPB1* and *RPB2* (Figure S1) showed a lack of separation in *S. bayanus* vs. *S. pastorianus (B)* and in *S. cerevisiae* vs. *S. pastorianus (C)*, with more resolution in the latter group when using *RPB2* than *RPB1* (Figure 6b vs. Figure 6a).

The *Candida* species were totally resolved with both markers (Figure 6c,d). A global comparison of *MeTRe* values showed that, in the *Saccharomyces* model, the four markers showed maximum values around 14 (*ACT1*), 80 (*TEF1a*), 88 (*RPB1*) and 78 (*RPB2*). These four values were, respectively, 270, 100, 300 and 330 in the *Candida* model. These data show the potential of *MeTRe* in evaluating the species separation (larger in *Candida* than in *Saccharomyces*) and in measuring the efficacy of the markers. *RPB1* and *RPB2* were very efficient in both taxonomic models, whereas *ACT1* had poor discrimination in *Saccharomyces* compared to the one in *Candida*. Moreover, all the four single-copy genes obtained from the genomes behaved much better than *ITS* and *LSU*, regardless of the source of these last sequences (compare Figure 2 and Figure 3 with Figure 5 and Figure 6). The method proposed for sequence retrieval returns the number of sequences homologous to the query. Whereas *ITS* and *LSU* are multicopy and could represent a family of paralogs, from the pipeline results, the single-copy genes appeared to have only one output. A possible exception could be *TEF2*, but with our settings, it was not returned, using *TEF1-α* as a probe. The absence of paralogs is a further advantage of these protein-encoding genes as markers that is expected to prevent the typical problems of paralogous genes taking different evolutionary trajectories, therefore producing contrasting results.

## 4. Discussion

The taxonomic analysis of a species based on marker sequences relies primarily on the distances among strains as the first step for tree reconstruction and for the application of thresholds, normally accepted as species delimitations. In seminal works introducing the marker sequence as a taxonomic tool, thresholds were generally described as the distance below which the strains are supposedly part of the same species [6,8,11]. In terms of species description, this definition should be considered as the maximum allowable distance between the two furthest strains of the same species, without considering any reference or type strains that should be well inside the species distribution. As a matter of fact, this application of the threshold is rather difficult to apply in the identification routine when an unknown strain must be associated to a known species. In fact, the addition of the strain to identify could change the species dimensions, and the researcher should therefore evaluate if the species with this new strain complies with the specifications that the maximum distance among all strains of the species has within the threshold. This procedure is obviously cumbersome and would require the availability of dedicated databases able to rapidly calculate the maximum distance within the species upon each strain addition. A much simpler approach, and closer to the spirit of the use of the type specimen concept, is to compare the distance of the unknown strain with the type strain of the species. This method only requires the sequences of type strains. The problem is that it is not reliable if the type strain is not central within the species distribution. This should not happen if the rules described above are strictly followed and if all sequence markers equally or linearly reflect the evolution of the genome. We know that this is not the case and that the centrality of the type strains is often not respected, nor, and even less, is the centrality of some of the markers. The centrality of a strain can be defined as the distance from the center of the species distribution, as suggested elsewhere [34]. The distance is calculated with the best available descriptors, as markers, but in the future, it could be the whole genome. Using this approach, it has been shown that most type strains of known yeast species are “central” [16]. The question on whether it is due to an effective “centrality” of the type or to other factors is a matter of discussion, considering that the type is not designed in the nomenclature code to be a reference, as stated by the Melbourne Nomenclature Code, “A nomenclatural type (typus) is that element to which the name of a taxon is permanently attached, … The nomenclatural type is not necessarily the most typical or representative element of a taxon”, https://www.iapt-taxon.org/historic/2012.htm. In any case, in the current taxonomic practice, the type is an anchor and, as such, prevents the species from moving away in the taxonomic space.

The use of fixed thresholds throughout the whole nomenclature relays the hypothesis that all species are of the same size, which is all but evident. A nominalist approach to species delimitation would impose that species are all similar or identical in size, and a fixed threshold could be used. Although this is not the place for a specific discussion on this topic, the acceptance of nominalism would seemingly leave no space for any claim on the correspondence between nomenclature and natural order [35]. Taxonomy should try to make both fit as close as possible, whereas the nomenclature may impair this goal by forcing the anchoring to the type and for stability reasons. The problems in species delimitation and strain identification [15] suggest either to collapse the species in a sort of continuum [36,37] or to find new metrics able to avoid the specific problems posed by distances and able to take into account the actual species internal variability.

On the other hand, the extensive use of NGS for both genomics and metagenomics [2,3,38,39] calls for the quantitative analysis of genes that could be retrieved from the genomes for strain identification and species delimitation or from metagenomic data for diversity estimation.

In both respects, *MeTRe* seems to match both requirements, because it can be used reversibly to compare the level of species separation and to evaluate the marker efficacy. The fact that *MeTRe* is nondimensional and that it considers the internal variability of the species (or of the strain set used) are factors allowing to use it in a generalized way. *MeTRe* allows an immediate definition of the separation between species simply by considering if their *MeTRe* are below (no separation) or over (separation) 1. Conversely, when different markers should be analyzed and compared, *MeTRe* gives the information on the minimum and maximum levels of separation and displays which species are or are not poorly separated. *MeTRe* was proposed in this paper with the specific intention of comparing the efficacy of single-copy markers from genomes with traditional rRNA markers sequences obtained with both the amplicon-based approach and those retrieved from genomes. The single-copy protein genes outperformed the rRNA markers to a large extent, suggesting that their usage in genomics and metagenomics is promising. The present analysis was limited by the number of strains’ genomes that contained all the analyzed genes. rRNA markers are not always present or well-assembled [38,40,41]; however, a more extensive analyses of these markers will be necessary to confirm the preliminary findings of the current study.

## 5. Conclusions

In conclusion, this paper showed the importance of considering not only the distances between the species but, also, within them, although a relatively small number of sequences could be considered for this proof of concept. Large scale analyses are currently carried out in our laboratories to apply this approach to the current yeast taxonomy with *MeTRe* and other indexes. At the same time, it was demonstrated that the single-copy protein-encoding genes from genomes ensure a good level of resolution, equal if not better than that achieved with standard rRNA markers. This paves the way not only to a more extended use of these markers in shotgun metagenomics but, also, to NGS-based approaches using an extended multi-marker barcode.

## Figures and Tables

**Table 1:** List of *Saccharomyces* and *Candida* genomes used for the analysis.

***S. bayanus***		***S. cerevisiae***		***S. kudriavzevii***	
GCA_001298625.1		GCA_003086655.1		GCA_000167075.2	
GCA_001515405.2		GCA_004328465.1		GCA_000256825.1	
GCA_003327605.1		GCA_000662435.2		GCA_000257025.1	
GCA_013180675.1		GCA_000976845.3		GCA_000256985.1	
GCA_013180065.1		GCA_000977385.2		GCA_900682665.1	
GCA_013180125.1		GCA_000977715.4		GCA_000257045.1	
GCA_013180165.1		GCA_003275125.1		GCA_000256845.1	
GCA_013180695.1		GCA_002571405.2		GCA_000257085.1	
		GCA_003274825.1		GCA_000257105.1	
		GCA_009738405.1		GCA_003327635.1	
***S. pastorianus***		***S. uvarum***		***S. paradoxus***	
GCA_001515445.2		GCA_000167035.1		GCA_002079055.1	
GCA_011022315.1		GCA_013265775.1		GCA_004353035.1	
GCA_013180355.1		GCA_013179955.1		GCA_004353095.1	
GCA_013180735.1		GCA_013180055.1		GCA_004353105.1	
GCA_013179865.1		GCA_013180345.1		GCA_000166955.1	
GCA_000805465.1		GCA_013179815.1		GCA_004352945.1	
GCA_001515425.2		GCA_013265705.1		GCA_004352955.1	
GCA_001483335.1		GCA_013180195.1		GCA_004352965.1	
GCA_001640265.1		GCA_013180235.1		GCA_009805645.1	
GCA_003004515.1		GCA_013179965.1		GCA_002079145.1	
***C. albicans***	***C. auris***		***C. glabrata***		***C. metapsilosis***
GCA_000182965.3	GCA_003013715.2.		GCA_000002545.2		GCA_008904905.1
GCA_002837675.1	GCA_008275145.1		GCA_002219185.1		GCA_900069165.1
GCA_003454735.1	GCA_014217455.1		GCA_002219195.1		
GCA_005890765.1	GCA_014673535.1		GCA_010111755.1		
***C. orthopsilosis***	***C. parapsilosis***		***C. tropicalis***		
GCA_000304155.1	GCA_000982555.2		GCA_000633855.1		
GCA_000315875.1	GCA_011316035.2		GCA_002864075.1		
GCA_004334915.1	GCA_014049445.1		GCA_006942135.1		
GCA_900002835.2	GCA_014049495.1		GCA_013177555.1		

**Table 2:** List of probe sequences used in the current study. ITS: Internal Transcribed Spacer and LSU: Large ribosomal subunit.

Species	Marker Sequences		
	***ACT1***	***ITS***	***LSU***
*C. albicans*	AJ389057	AB032172	U45776
*C. auris*	AJ389073	AB375772	AB375773
*C. glabrata*	AJ389073	AY046165	U44808
*C. metapsilosis*	AJ508485	FJ872019	AY497667
*C. parapsilosis*	AJ508485	KP054272	U45754
*C. orthopsilosis*	AJ508485	FJ872018	FJ746056
*C. tropicalis*	AJ508499	AF287910	U45749
*S. cerevisiae*	AJ389075	AY046146	AY048154
	***RPB1***	***RPB2***	***TEF1-α***
*C. albicans*	JQ713048	XM_713079.2	AF402066
*C. auris*	MK294611.1	XM_029033121.1	AF402029
*C. glabrata*	AY497705	AF527898	AF402029
*C. metapsilosis*	LN680790.1:15517-16901	LN680773.1: 56482-58821	LN680790.1:1502560-1503683
*C. parapsilosis*	XM_714321.2	JQ698980	AF402066
*C. orthopsilosis*	LN680790.1:15517-16901	LN680773.1: 56482-58821	LN680790.1:1502560-1503683
*C. tropicalis*	CP017630.1:2260358-2265544	CP017623.1:319665-323369	AF402066
*S. cerevisiae*	JQ713023	JQ698955	AF402004

## Data Availability

No new data were created or analyzed in this study.

## Supplementary Materials

The following are available online at https://www.mdpi.com/2076-2607/9/2/299/s1. Figure S1: *MeTRe* among and within the Saccharomyces and Candida species. Legend: Resolution calculated for the genomic *RPB1* and genomic *RPB2* of Saccharomyces are displayed in panels (a,b), respectively. Genomic *RPB1* and genomic *RPB2* of Candida are reported in panels (c,d). Each column shows the resolution of the species positioned in a red line (reported above each column) from the other species under analysis. The species placed below the red line have no resolution with the species reported above each column. Table S1: GenBank Accession numbers for the ITS and LSU sequences of the *Saccharomyces sensu stricto* complex and *Candida* genus, used for comparison with the respective sequences retrieved from the genomes.
