# Supplementary figures and images for: What Is the Best Lens? Comparing the Resolution Power of Genome-Derived Markers and Standard Barcodes
Source: Microorganisms. 2021 Feb 2;9(2):299. doi: 10.3390/microorganisms9020299 (PMC7912933; doi:10.3390/microorganisms9020299)

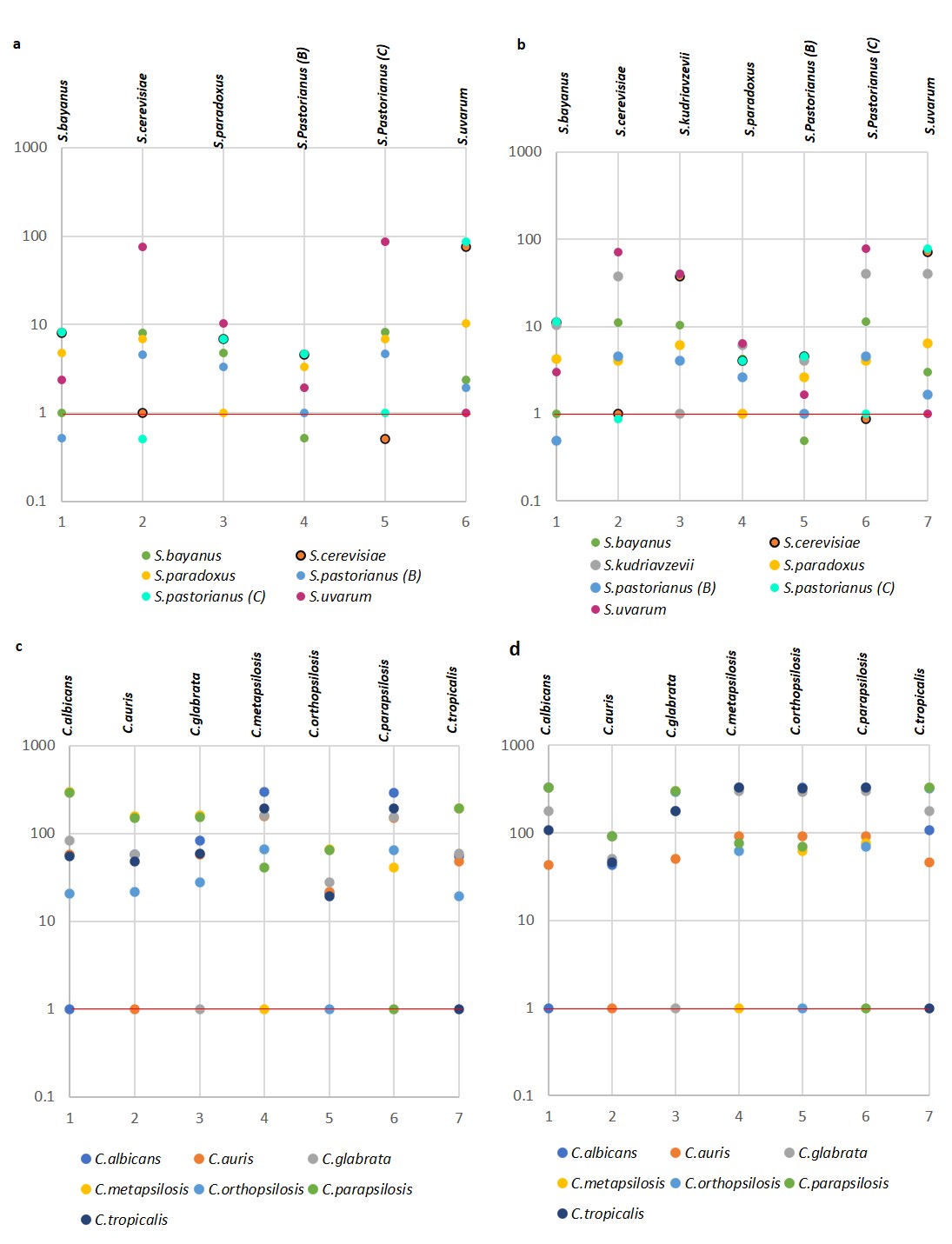

Supplement: Supplementary file 1 [file microorganisms-09-00299-s001.zip › Figure S1.docx]
